# Isoalantolactone induces apoptosis through ROS-mediated ER stress and inhibition of STAT3 in prostate cancer cells

**DOI:** 10.1186/s13046-018-0987-9

**Published:** 2018-12-12

**Authors:** Wei Chen, Ping Li, Yi Liu, Yu Yang, Xueting Ye, Fangyi Zhang, Hang Huang

**Affiliations:** 1 Department of Urology, |The First Affiliated Hospital of Wenzhou Medical University, Wenzhou, 325035 Zhejiang China; 2 Department of Gynaecology and Obstetrics, The Second Affiliated Hospital of Wenzhou Medical University, Wenzhou, Zhejiang China

**Keywords:** Reactive oxygen species, Isoalantolactone, ER stress, STAT3, Prostate cancer

## Abstract

**Background:**

Prostate cancer is one of the most commonly diagnosed cancers in men worldwide. Currently available therapies for metastatic prostate cancer are only marginally effective. Therefore, new therapeutic agents are urgently needed to improve patient outcome. Isoalantolactone (IATL), an active sesquiterpene naturally present in many vegetables and medicinal plants, is known to induce cell death and apoptosis in various cancer cell lines. Nevertheless, antitumor mechanisms initiated by IATL in cancer cells have not been fully defined.

**Methods:**

Cell apoptosis and cellular ROS levels were analyzed by flow cytometry. Western blot and qRT-PCR were used to analyze the protein and mRNA levels of indicated molecules, respectively. Nude mice xenograft model was used to test the effects of IATL on prostate cancer cell growth in vivo.

**Results:**

In this study, we found that IATL dose-dependently inhibited cancer cell growth and induced apoptosis in PC-3 and DU145 cells. Mechanistically, our data found that IATL induced reactive oxygen species (ROS) production, resulting in the activation of endoplasmic reticulum stress pathway and eventually cell apoptosis in prostate cancer cells. IATL also decreased the protein expression levels of p-STAT3 and STAT3, and the effects of IATL were reversed by pretreatment with N-acetyl-L-cysteine (NAC). In vivo, we found that IATL inhibited the growth of prostate cancer xenografts without exhibiting toxicity. Treatment of mice bearing human prostate cancer xenografts with IATL was also associated with induction of ER stress and inhibtion of STAT3.

**Conclusion:**

In summary, our results unveil a previously unrecognized mechanism underlying the biological activity of IATL, and provide a novel anti-cancer candidate for the treatment of prostate cancer.

## Background

Prostate cancer is one of the most commonly diagnosed cancers in men worldwide, with 1.1 million new cases of prostate cancer were estimated to have occurred in 2012 [1]. Current prostate cancer therapy includes surgery, androgen deprivation therapy, chemotherapy, and radiation [2]. Although androgen deprivation therapy is effective in the first few years of treatment, most patients eventually develop resistance to this therapy and progress into castration resistant prostate cancer (CRPC) [3]. On the other hand, the existing conventional chemotherapy usually leads to severe side effects and drug resistance in prostate cancer patients [4, 5]. Therefore, new therapeutic agents are urgently needed to improve therapeutic outcome in prostate cancer patients.

Natural products have been historically recognized as invaluable sources of inspiration for the development of new drugs [6]. An assessment of all U.S. Food and Drug Administration (FDA) approved new molecular entities indicated that natural products and their derivatives represent more than one-third of all FDA-approved new molecular entities, especially for anticancer molecules, which are significantly enriched with natural products [7]. Isoalantolactone (IATL), an active sesquiterpene naturally present in many vegetables and medicinal plants, was recently identified as selectively toxic to cancer cells [8, 9]. The pleiotropic anticancer efects of IATL have also been demonstrated in diverse malignancies including esophageal cancer, lung carcinoma, breast cancer and pancreatic cancer [9–12]. More interestingly, IATL have also been reported to exert anti-proliferative and pro-apoptotic effects against prostate cancer cells [13], but the in depth molecular mechanisms of its anticancer effects were not deciphered in this study.

In the present study, we have examined the effects of IATL on prostate cancer cells in vitro and in human prostate cancer xenografts. We found that IATL inhibited cell proliferation and induced cell apoptosis in prostate cancer cells. Mechanistically, we present evidence for the first time that ER stress contributes to IATL-induced apoptosis in prostate cancer cells. We also investigated the underlying mechanism of cytotoxic effects of IATL to confirm the upstream regulator of ER stress in the apoptotic process. Our results unveil a previously unrecognized mechanism underlying the biological activity of IATL, and provide a novel anti-cancer candidate for the treatment of prostate cancer.

## Methods

### Reagents and cell culture

Isoalantolactone (Tauto Biotech, Shanghai, China) was suspended in dimethyl sulfoxide and stored at − 20 °C. N-acetyl-L-cysteine (NAC) and catalase were purchased from Sigma (St. Louis, MO, USA). Antibodies including anti-GAPDH, m-IgGκ BP-HRP and mouse anti-rabbit IgG-HRP were purchased from Santa Cruz Biotechnology (Santa Cruz, CA, USA). Antibodies including anti-CHOP, anti-ATF4, anti-p-eIF2α, anti-eIF2α, anti-Bcl-2 and anti-cle-caspase-3 were purchased from Cell Signaling Technology (Danvers, MA, USA). Antibodies including anti-p-STAT3 and anti-STAT3 were purchased from Abcam (Cambridge, MA, USA). FITC Annexin V apoptosis Detection Kit I and Propidium Iodide (PI) were purchased from BD Pharmingen (Franklin Lakes, NJ, USA). Human prostate cancer cell lines PC-3 and DU145 were purchased from the Institute of Biochemistry and Cell Biology, Chinese Academy of Sciences. The cells were maintained in RPMI 1640 medium containing 10% fetal bovine serum (FBS) at 37 °C in a humidified incubator with an atmosphere of 5% CO_2_.

### Cell viability assay

To assess viability following IATL treatment, cells were seeded on 96-well plates at a density of 8 × 10^3^ per well, and allowed to attach overnight in complete growth medium. IATL was dissolved in DMSO and diluted with 1640 medium to final concentrations of 2.5, 5, 10, 20, 30, 40, 50 and 60 μM. Prostate cancer cells were incubated with IATL for 24 h before the MTT assay.

### Cell apoptosis analysis

Cell apoptosis analysis was performed as described previously [14]. Briefly, cells were treated with IATL for 24 h. Then the cells were harvested, washed twice with ice-cold PBS. The washed cell samples were resuspended in 500 μL binding buffer, and evaluated for apoptosis by double staining with Annexin V and Propidium Iodide (PI) in binding buffer for 30 min using a FACSCalibur flow cytometer.

### Western blot analysis

Cells or tumor tissues were homogenized in protein lysate buffer, and debris was removed by centrifugation at 12,000 g for 10 min at 4 °C. Concentrations of protein in whole-cell extracts were determined using the Bradford protein assay (Bio-Rad, CA, USA). The same amount of lysate proteins were separated by electrophoresis on SDS-polyacrylamide gels, and electroblotted onto polyvinylidene difluoride membrane. The blots were blocked for 2 h at room temperature with fresh 5% non-fat milk in TBST and then incubated with specific primary antibody in TBST overnight at 4 °C. Following three washes with TBST, the blots were incubated with horseradish peroxidase-conjugated secondary antibodies for 1 h, and the immunoreactive bands were visualized by using ECL kit. The density of the immunoreactive bands was analyzed using Image J computer software.

### Measurement of reactive oxygen species generation

Cellular ROS contents were measured by flow cytometry. Briefly, cells were plated on 6-well plates, and allowed to attach overnight in complete growth medium. Cells were then exposed to IATL for the indicated times. Following treatments, cells were stained with 10 μM DCFH-DA (Beyotime, Shanghai, China) at 37 °C for 30 min in the dark. Cells were collected and the fluorescence was analyzed by FACSCalibur flow cytometer. In some experiments, cells were pretreated with 5 mM NAC for 2 h prior exposure to IATL and analysis of ROS generation.

### Quantitative RT-PCR

Cells were harvested and total RNA was extracted from the cells using the Trizol reagent according to the manufacturer’s instructions (Invitrogen, CA, USA). Both reverse transcription and quantitative PCR were carried out using a two-step M-MLV Platinum SYBR Green qPCR SuperMix-UDG Kit (Invitrogen, CA, USA). Eppendorf Mastercycler eprealplex detection system (Eppendorf, Hamburg, Germany) was used for q-PCR analysis. The following gene-specific primer pairs were used: CHOP: (F) 5′-atggcagctgagtcattgcctttc-3′, (R) 5′-agaagcagggtcaagagtggtgaa-3′. β-actin: (F) 5′-ttcctgggcatggagtcct-3′, (R) 5′-aggaggagcaatgatcttgatc-3′. Gene expressions were analyzed with the comparative threshold cycle method after normalizing to the housekeeping gene β-actin.

### Transient transfection of small interfering RNA (siRNA)

The siRNA duplexes used in this study were purchased from Invitrogen (Carlsbad, CA, USA) and have the following sequences: CHOP (5’-GAGCUCUGAUUGACCGAAUGGUGAA-3′). Negative Universal Control (Invitrogen, CA, USA) was used as the control. Cells were seeded on 6-well plates and cultured for 24 h in complete growth medium, and then were transfected with siRNA duplexes against human CHOP (100 nM) or control siRNA by lipofectamine 3000 (Invitrogen, CA, USA) according to manufacturer’s instructions.

### Determination of caspase-3/9 activity

Caspase-3/9 activity in cell lysates was determined using a caspase-3/9 activity kit (Beyotime, Shanghai, China) according to the manufacturer’s protocol. Caspase-3/9 activity was normalized by the protein concentration of the corresponding cell lysate and expressed as percentage of treated cells to that of control.

### In vivo antitumor study

Athymic nude mice (nu/nu, 4–5 week, male) were used for in vivo experiments. All animals were handled according to the Institutional Animal Care and Use Committee (IACUC) guidelines, Wenzhou Medical University. The animals were randomly divided into various groups. Animals were housed at a constant room temperature with a 12 h light/12 h dark cycle and fed a standard water and rodent diet. Cells were harvested and injected subcutaneously (5 × 10^6^ cells in 100 μL of PBS) into the right flank of mice. Mice were treated with IATL at the dose of 10 mg/kg body weight by intraperitoneal (i.p.) injection once every other day of induction. At the end of experiment, the animals were sacrificed and the tumors were removed and weighed for use in proteins expression studies. The tumor volumes were determined by measuring length (l), width (w) and calculating volume (V = 0.5 × l × w^2^) at the indicated time points.

### HE staining

For histologic analysis, the harvested liver and kidney tissues of mice were fixed in 4% paraformalclehyde, dehydrated with an ethanol gradient, embedded in paraffin, and the paraffin tumor tissue sections (5 μm) were stained with hematoxylin and eosin. Each image of the sections was captured using a light microscope (Nikon, Japan).

### MDA assay

The tissue samples were homogenized and sonicated in RIPA buffer on ice. Tissue lysates were then centrifuged at 12,000 g for 10 min at 4 °C to collect the supernatant. The total protein concentrations were determined using the Bradford protein assay. Tumor tissue proteins were normalized according to their concentrations and subjected to MDA assay as described in the Lipid Peroxidation MDA assay kit (Beyotime, Shanghai, China). MDA levels were detected using multimode microplate readers (SpectraMax M5, Molecular Devices, USA) at 532 nm.

### Statistical analysis

All experiments were assayed in triplicate. Data are expressed as means ± SEM. All statistical analyses were performed using GraphPad Pro. Prism 5.0 (GraphPad, SanDiego, CA). Student’s t-test and two-way ANOVA were employed to analyze the differences between data sets. A *p* value < 0.05 was considered statistically significant.

## Results

### IATL inhibits cells growth and induces apoptosis in prostate cancer cells

**Fig. 1:**
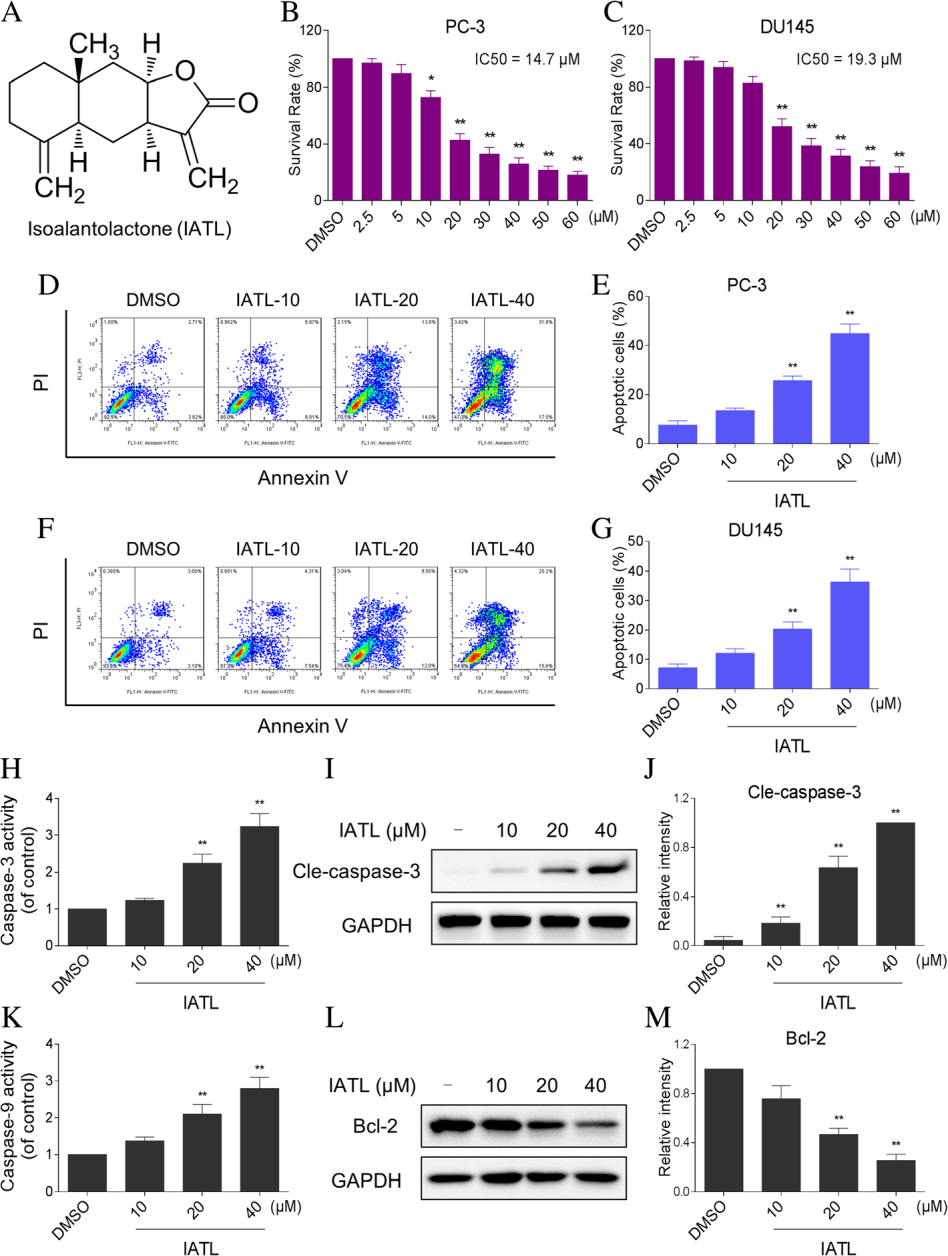
IATL suppresses cells growth and induces apoptosis in prostate cancer cells. **a** The chemical structure of IATL. **b**-**c** PC-3 and DU145 cells were incubated with increasing doses of IATL (2.5–60 μM) for 24 h respectively. Cell viability was determined by MTT assay. **d**-**g** PC-3 or DU145 cells were incubated with IATL for 24 h, percentage of cell apoptosis was determined by Annexin-V/PI staining and flow cytometry. **h** Cells were incubated with IATL for 20 h, caspase-3 activity in the cell extracts were determined by an assay kit using specific substrate. **i**-**j** Cells were incubated with IATL for 20 h, the protein level of cle-caspase-3 was determined by western blot. The results shown are representative of at least three independent experiments. **k** Cells were incubated with IATL for 20 h, caspase-9 activity in the cell extracts were determined by an assay kit using specific substrate. **l**-**m** Cells were incubated with IATL for 20 h, the protein level of Bcl-2 was determined by western blot. The results shown are representative of at least three independent experiments

To explore the effects of IATL on the growth of prostate cancer cells, two human prostate cancer cell lines, PC-3 and DU145 cells were treated with IATL at different concentrations (0–60 μM) for 24 h. As show in Fig. 1b-c, IATL treatment decreased the viability of PC-3 and DU145 cells in a dose-dependent manner. We next analyzed the potential of IATL to induce apoptosis in PC-3 and DU145 cells. As shown in Fig. 1d-g, treatment with IATL for 24 h dose-dependently increased the proportion of apoptotic cells in both PC-3 and DU145 cells. The effects of IATL on caspase-3 activation were determined using caspase acitivity assay and western blot analysis. We found that IATL induced a significant increase in caspase-3 activity, and also elevated cleavage of caspase-3 in PC-3 cells (Fig. 1h-j). Notably, caspase-9 activity was also significantly elevated after IATL treatment in PC-3 cells (Fig. 1k). In addition, IATL treatment significantly suppressed the expression of Bcl-2, suggesting that mitochondrial pathway is involved in IATL-induced apoptosis in prostate cancer cells (Fig. 1l-m). Overall, these results demonstrate that IATL exhibits significant anti-cancer activity by inhibiting cell proliferation and inducing apoptosis in prostate cancer cells.

### IATL induces oxidative stress in prostate cancer cells

**Fig. 2:**
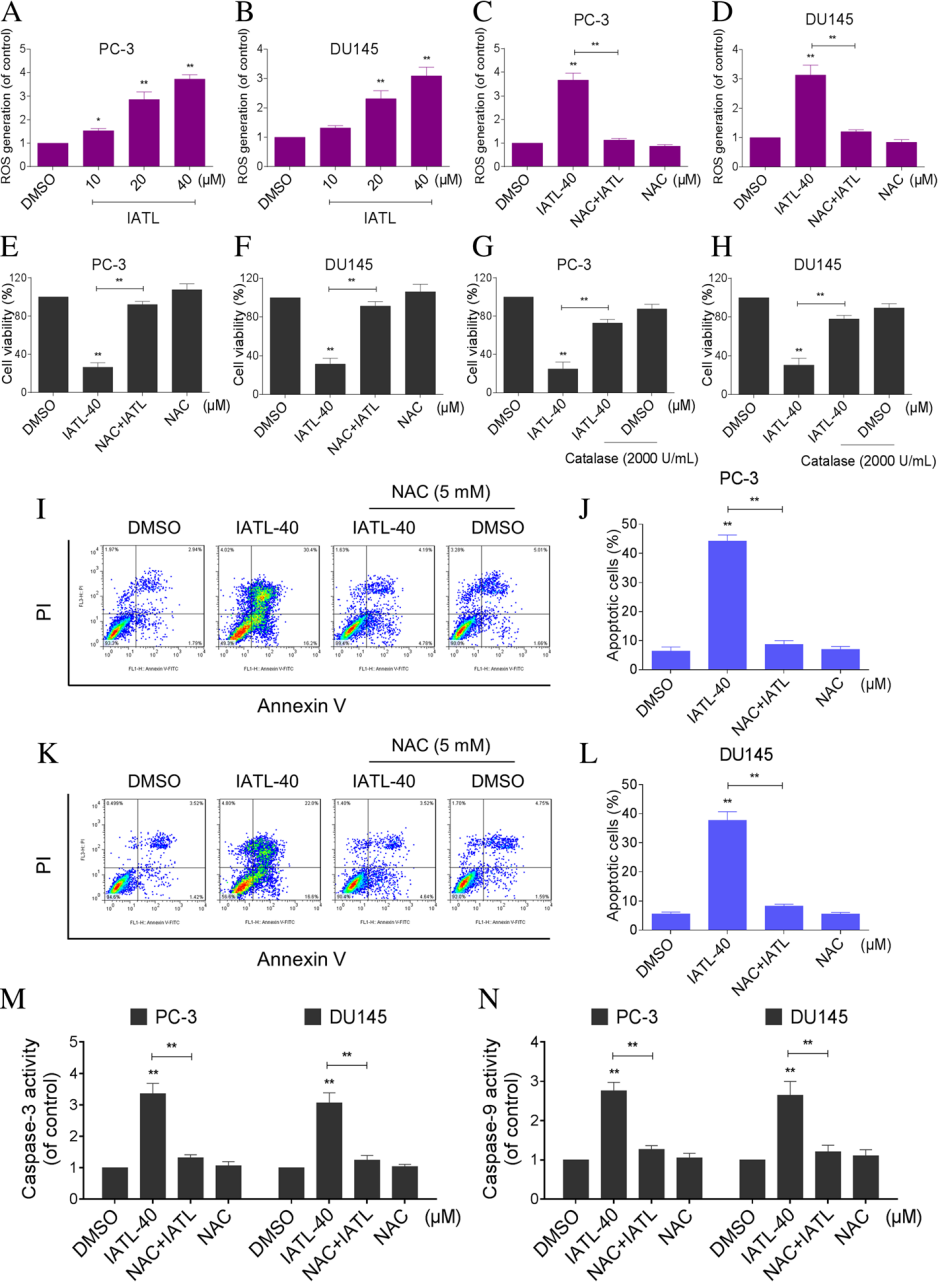
IATL induces oxidative stress in prostate cancer cells. **a**-**b** Intracellular ROS generation induced by increasing doses of IATL was measured in PC-3 or DU145 cells by staining with DCFH-DA (10 μM) and flow cytometry analysis. **c**-**d** Cells were pre-incubated with 5 mM NAC for 2 h before exposure to IATL. Intracellular ROS generation was measured by flow cytometry. **e**-**f** Cells were pre-incubated with 5 mM NAC for 2 h before exposure to IATL for 24 h, cell viability was determined by MTT assay. **g**-**h** Cells were pre-incubated with 2000 U/mL catalase for 2 h before exposure to IATL for 24 h, cell viability was determined by MTT assay. **i**-**l** Cells were pre-incubated with 5 mM NAC for 2 h before exposure to IATL for 24 h, percentage of cell apoptosis was determined by Annexin-V/PI staining and flow cytometry. The results shown are representative of at least three independent experiments. **m**-**n** Cells were pre-incubated with 5 mM NAC for 2 h before exposure to IATL for 20 h. Caspase-3 or caspase-9 activity in the cell extracts was determined by an assay kit using specific substrate

The generation of ROS has been reported to play an important role in the pro-apoptotic effect of IATL in some cancer cell lines [9, 11]. Therefore, we measured the intracellular ROS levels in IATL-treated cells by flow cytometry. As shown in Fig. 2a-b, IATL treatment caused a dose-dependent increase in ROS levels in PC-3 and DU145 cells. To investigate the role of ROS in mediating IATL’s anti-cancer effects, ROS scavenger N-acetyl-L-cysteine (NAC) was used. As shown in Fig. 2c-d, pretreatment with NAC significantly reversed the IATL-induced increase in ROS levels as expected. The MTT results revealed that scavenging of ROS markedly attenuated IATL-induced cell growth inhibition against prostate cancer cells (Fig. 2e-f). To further determine the ROS involved in the IATL-induced cell growth inhibition against prostate cancer cells, a non-thiol antioxidant catalase was used. As shown in Fig. 2g-h, pretreatment with catalase for 2 h significantly reversed IATL-induced cell death in PC-3 and DU145 cells. Additionally, NAC pretreatment fully reversed IATL-induced cell apoptosis in PC-3 and DU145 cells (Fig. 2i-l). Meanwhile, the activation of caspase-3 and caspase-9 was also reversed by NAC pretreatment (Fig. 2m-n). These results demonstrate that ROS generation is the key regulator of IATL-induced apoptosis in prostate cancer cells.

### ER stress contributes to IATL-induced apoptosis in prostate cancer cells

**Fig. 3:**
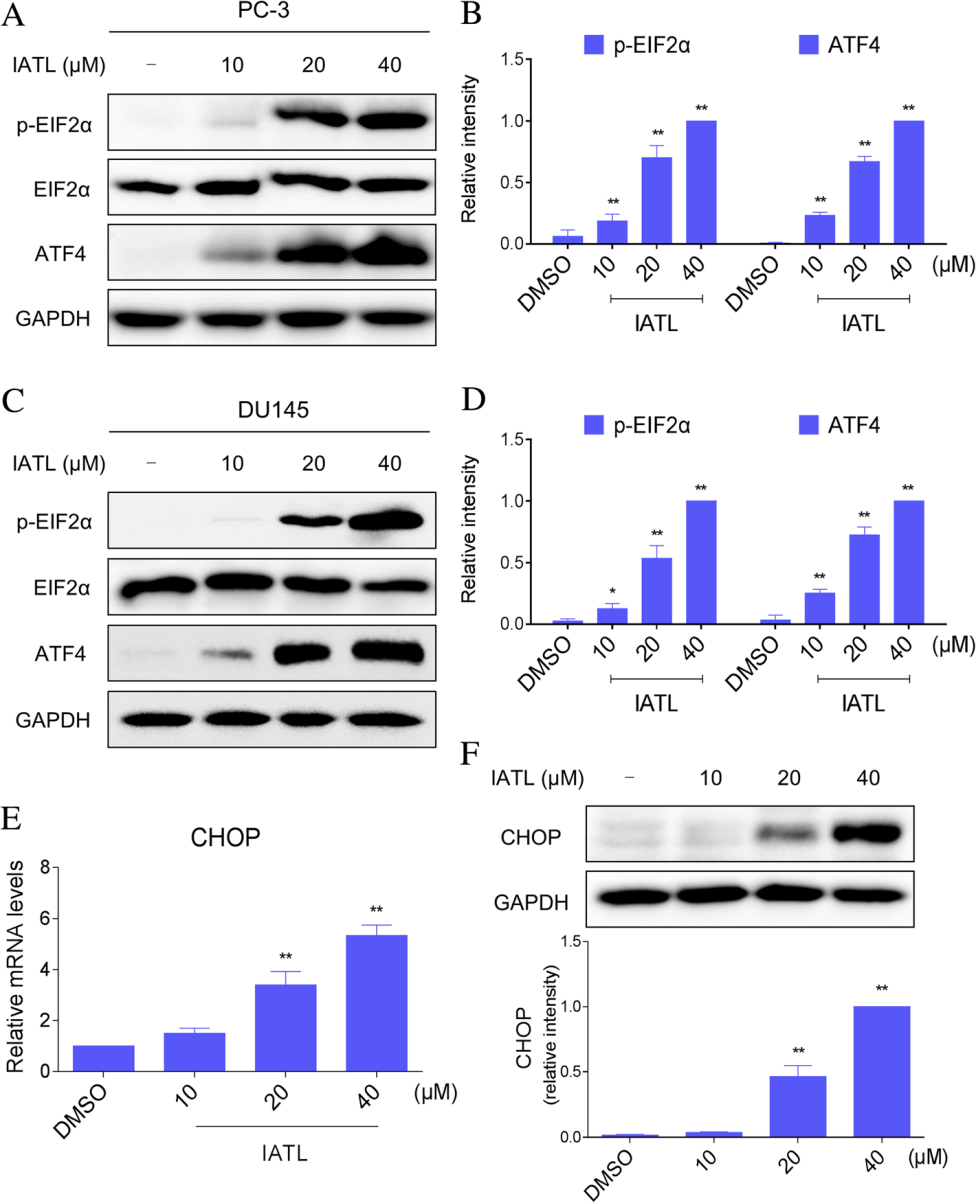
IATL activates ER stress pathway in prostate cancer cells. **a**-**b** PC-3 cells were treated with IATL for 4 h, the protein levels of p-eIF2α and ATF4 were determined by western blot. **c**-**d** DU145 cells were treated with IATL for 4 h, the protein levels of p-eIF2α and ATF4 were determined by western blot. **e** PC-3 cells were treated with IATL for 6 h. The mRNA expression of CHOP was analyzed by qRT-PCR. **f** PC-3 cells were treated with IATL for 12 h, the protein level of CHOP was determined by western blot. The results shown are representative of at least three independent experiments

Endoplasmic reticulum (ER) stress is emerging as a modulator of different pathologies and as an important mechanism contributing to cancer cell death in response to therapeutic agents [15, 16]. In several instances, oxidative stress and the onset of ER stress occur together [17, 18]. Therefore, we examined the expression of ER stress-related proteins, such as p-eIF2α and ATF4 in IATL-treated prostate cancer cells. Western blot analysis indicated that IATL treatment dose-dependently increased the expression of p-eIF2α and ATF4 in the PC-3 and DU145 cells (Fig. 3a-d). CHOP is known as a crucial factor that mediates ER stress-induced apoptosis [19]. We found that IATL treatment resulted in a significant increase in the mRNA and protein levels of CHOP (Fig. 3e-f).

**Fig. 4:**
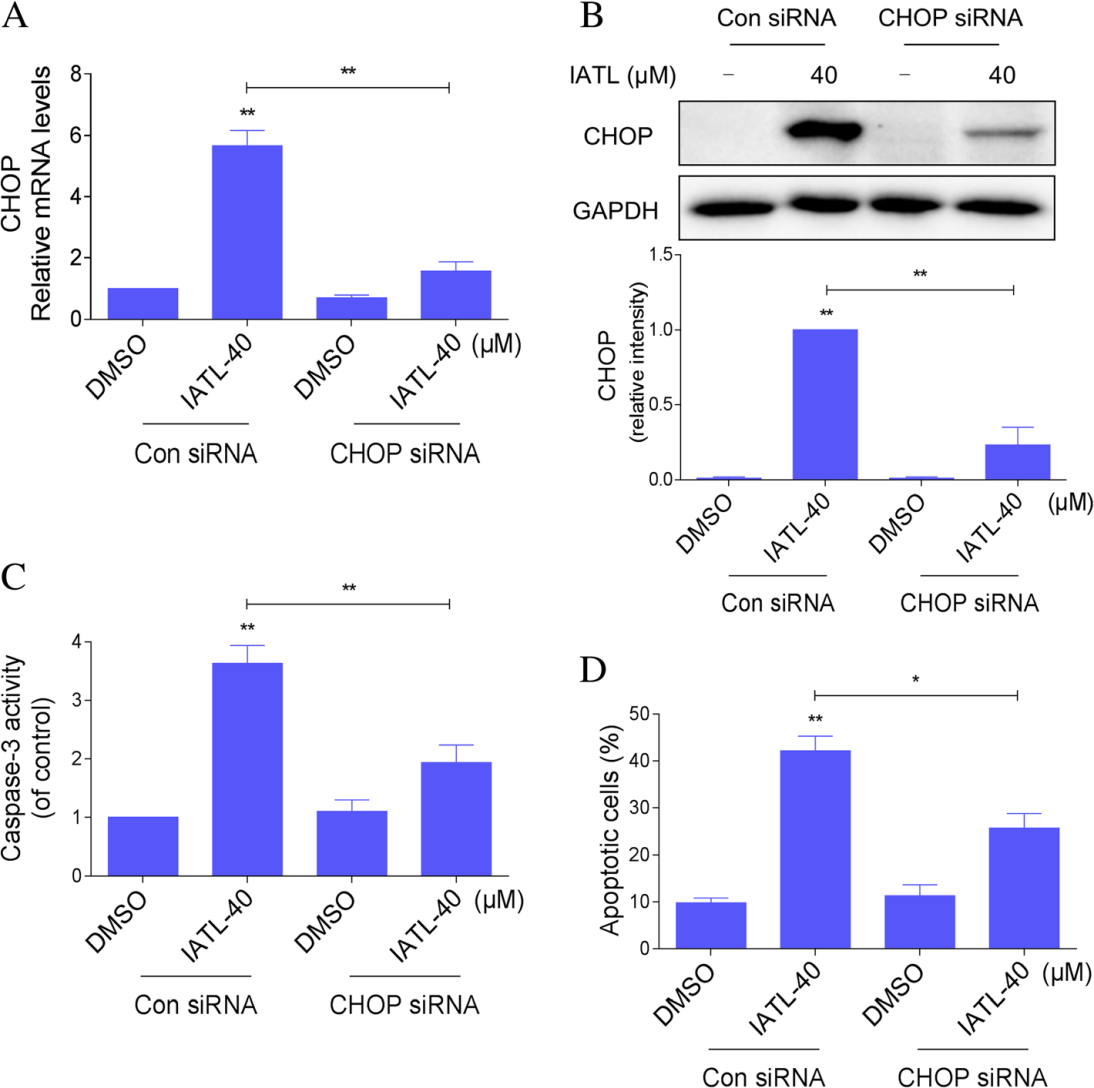
ER stress contributes to IATL-induced apoptosis in prostate cancer cells. **a** PC-3 cells were infected with CHOP siRNA or control siRNA, the mRNA expression of CHOP was analyzed by qRT-PCR after stimulation with IATL (40 μM) for 6 h. **b** PC-3 cells transfected with CHOP siRNA or control siRNA were treated with IATL (40 μM) for 12 h, the protein level of CHOP was determined by western blot. **c** PC-3 cells transfected with CHOP siRNA or control siRNA were treated with IATL (40 μM) for 20 h, caspase-3 activity in the cell extracts were determined by an assay kit using specific substrate. **d** PC-3 cells transfected with CHOP siRNA or control siRNA were treated with IATL (40 μM) for 24 h, percentage of cell apoptosis was determined by Annexin-V/PI staining and flow cytometry. The results shown are representative of at least three independent experiments

To further investigate whether ER stress was involved in the anti-cancer effects of IATL. We then examined the effect of siRNA-mediated depletion of CHOP in PC-3 cells. As shown in Fig. 4a-b, knockdown of CHOP via transfection of siRNA markedly attenuated CHOP expression in the mRNA and protein levels. This was associated with an appreciable reduction in IATL-induced caspase-3 activation and apoptosis in PC-3 cells (Fig. 4c-d). These findings demonstrate that IATL-induced cell apoptosis is, at least in part, mediated by activation of ER stress pathway.

### Induction of ER stress by IATL is dependent on ROS production in prostate cancer cells

**Fig. 5:**
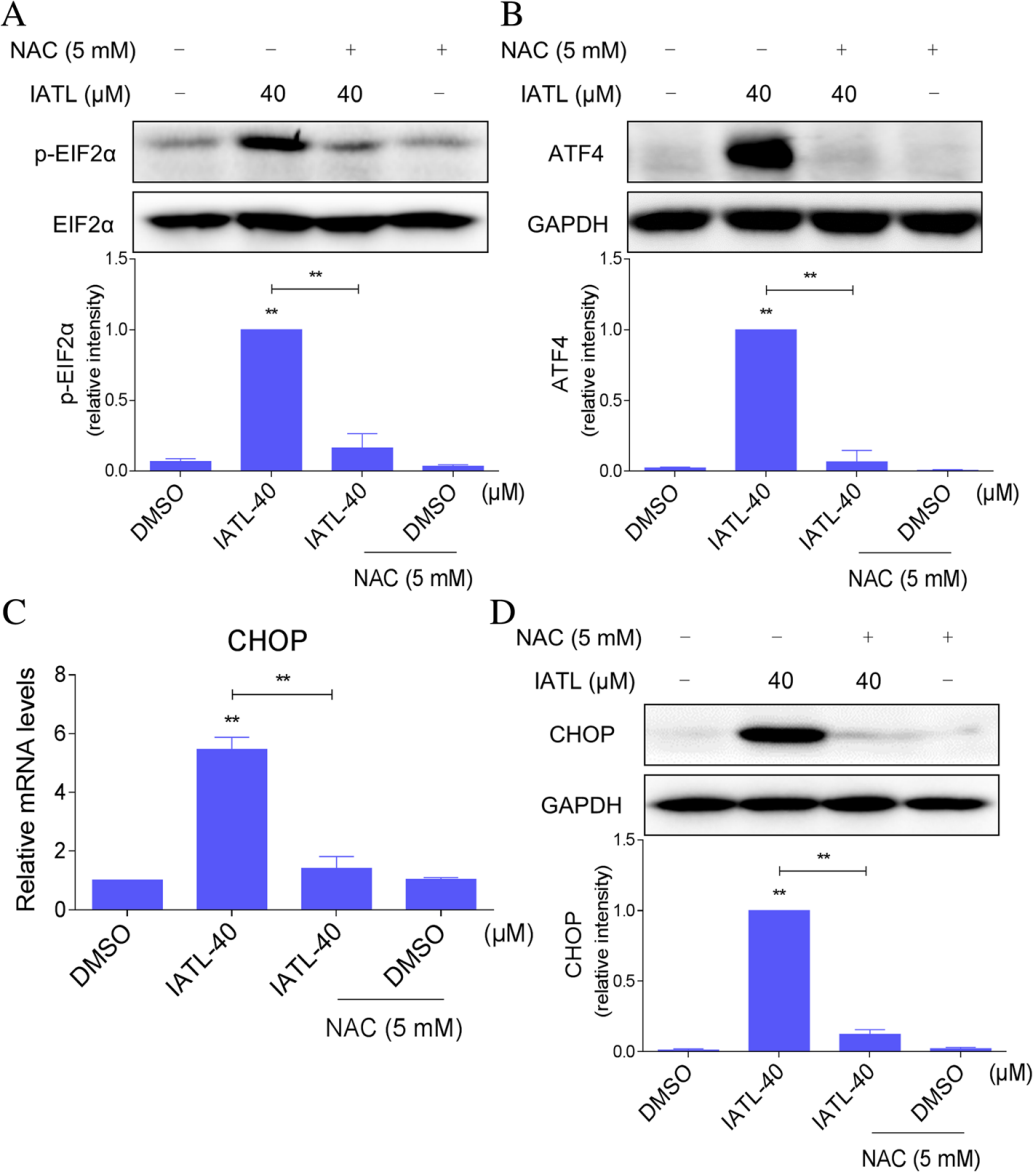
Induction of ER stress activation and cancer cell apoptosis by IATL are dependent on ROS production. **a**-**b** PC-3 cells were pre-incubated with or without 5 mM NAC for 2 h before exposure to IATL for 4 h. The protein levels of p-eIF2α and ATF4 were determined by western blot. **c** PC-3 cells were pre-incubated with or without 5 mM NAC for 2 h before exposure to IATL for 6 h. The mRNA expression of CHOP was analyzed by qRT-PCR. **d** PC-3 cells were pre-incubated with or without 5 mM NAC for 2 h before exposure to IATL for 12 h. The protein level of CHOP was determined by western blot. The results shown are representative of at least three independent experiments

We then tested the effect of NAC on IATL-induced ER stress. As shown in Fig. 5a-b, pretreatment with NAC significantly reversed the IATL-induced increase in p-eIF2α and ATF4 in PC-3 cells. In addition, the IATL-induced effects on CHOP expression in the mRNA and protein levels were attenuated by pretreatment with NAC (Fig. 5c-d). These findings suggest that ROS induction may be upstream regulator of IATL-induced ER stress.

### IATL inhibits STAT3 phosphorylation and expression in prostate cancer cells

**Fig. 6:**
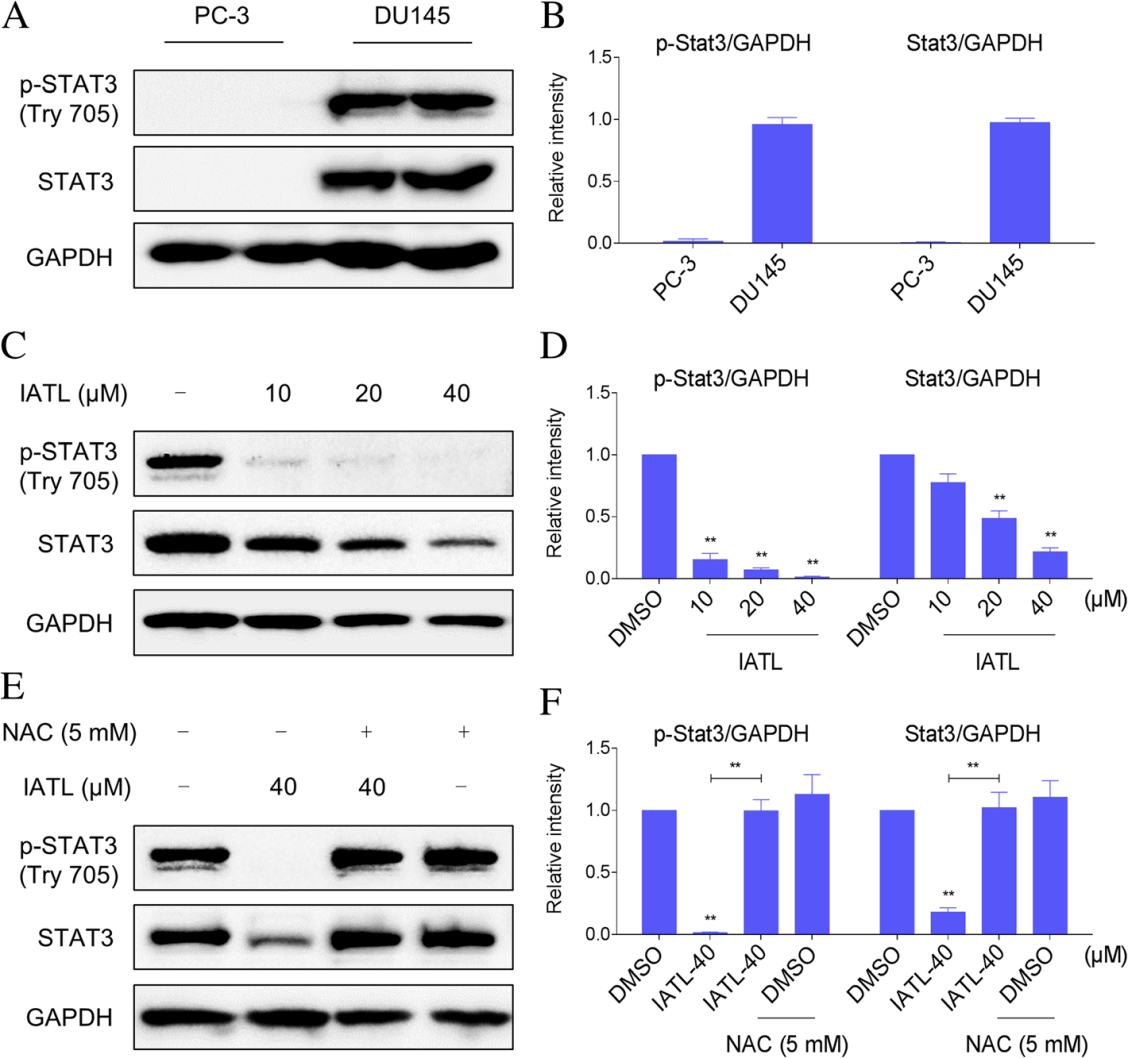
IATL inhibits STAT3 phosphorylation and expression in prostate cancer cells. **a**-**b** The levels of p-STAT3 and STAT3 in PC-3 and DU145 cells were detected by western blot. **c**-**d** DU145 cells were treated with IATL (10, 20, or 40 μM) for 12 h, the levels of p-STAT3 and STAT3 were detected by western blot. GAPDH was used as internal control. **e**-**f** DU145 cells were pre-incubated with or without 5 mM NAC before exposure to IATL (40 μM) for 12 h, the expression of p-STAT3 and STAT3 were detected by western blot. GAPDH was used as internal control. The results shown are representative of at least three independent experiments

Signal transducer and activator of transcription 3 (STAT3) is an oncogenic transcription factor that is constitutively activated in many types of solid tumors, including prostate cancer [20, 21]. We found that STAT3 is expressed in constitutively active form in DU145 cells, but was expressed at a very low level in PC-3 cells (Fig. 6a-b). To study whether IATL modulates STAT3 activation, DU145 cells were treated with IATL for different concentrations, and then STAT3 and STAT3 phosphorylation were examined by western blot. We found that constitutive p-STAT3 (Tyr 705) in DU145 cells was substantially reduced upon IATL treatment (Fig. 6c-d). Moreover, IATL also decreased the protein expression of STAT3, and the effects of IATL were reversed by pretreatment with NAC (Fig. 6e-f). These findings demonstrate a unique approach for targeting STAT3 by ROS-inducing anticancer agents.

### IATL inhibits DU145 xenograft tumor growth in vivo

**Fig. 7:**
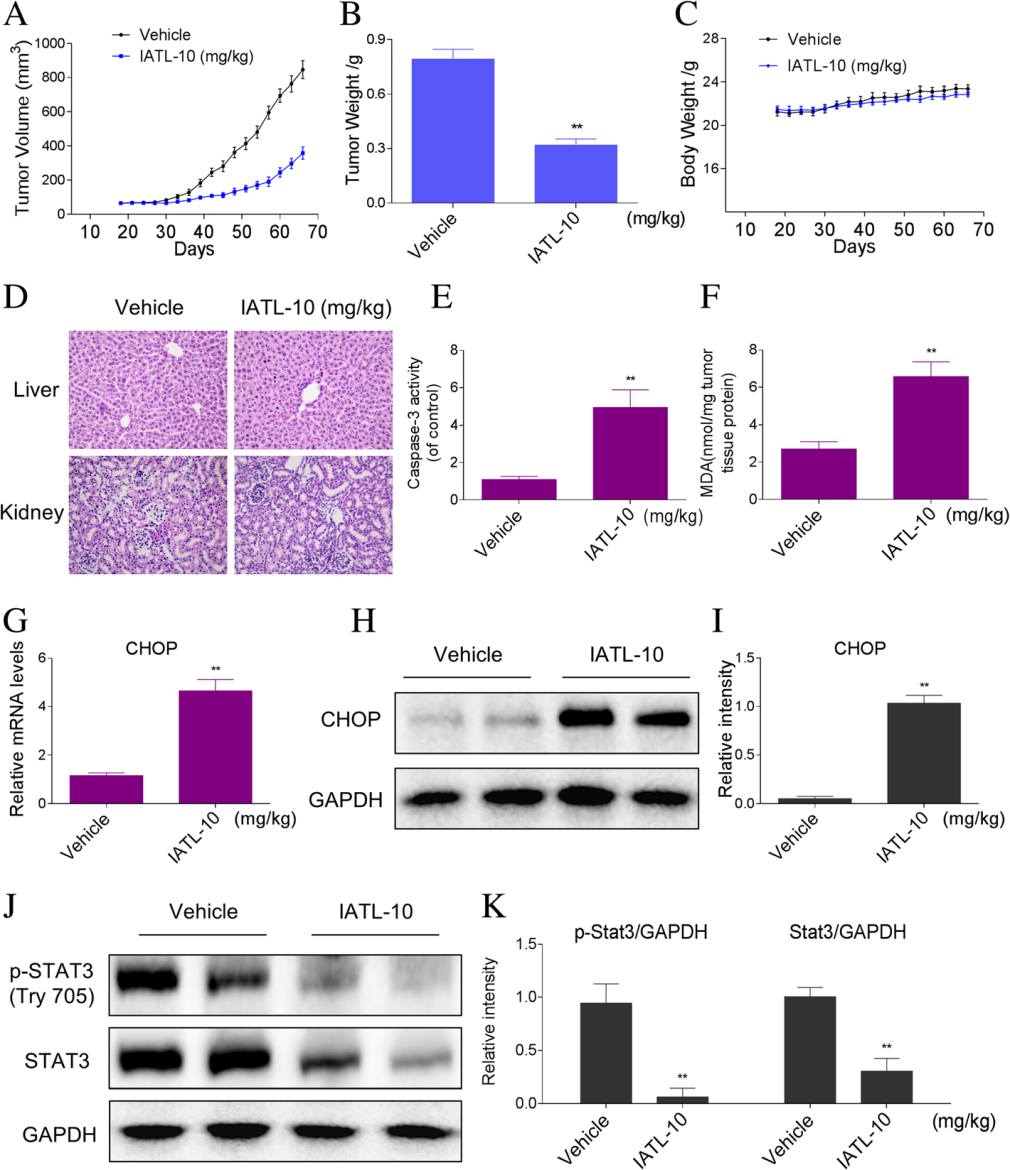
IATL inhibits DU145 xenograft tumor growth in vivo. **a**-**b** IATL treatment significantly inhibited tumor volume (**a**) and tumor weight (**b**) of DU145 human prostate cancer xenografts in nude mice, but did not affect body weight (**c**) of mice. **d** HE staining of the major organs, magnification 20x. **e** The levels of MDA in the tumor tissues. **f** Caspase-3 activity in the tumor tissues was determined by an assay kit using specific substrate. **g** The mRNA expression of CHOP in the tumor tissues. **h-i** Western blot analysis on the expression of CHOP from respective tumor tissue lysates. GAPDH was used as protein loading control. **j**-**k** Western blot analysis on the expression of p-STAT3 and STAT3 from respective tumor tissue lysates. GAPDH was used as protein loading control

To investigate the effects of IATL on tumor growth in vivo, we used a subcutaneous xenograft model of DU145 cells in immunodeficient mice. Intraperitoneal administration of IATL at dose of 10 mg/kg markedly reduced DU145 tumor volume and weight versus vehicle control (Fig. 7a-b). Importantly, IATL treatment for 49 days was well tolerated, did not lead to significant weight loss (Fig. 7c). The cytotoxic effect of IATL was evaluated by measuring histopathology of liver and kidney versus vehicle control. The results also revealed that IATL treatment did not result in observable toxicity (Fig. 7d). To determine whether the in vitro mechanisms, we identified are also at play in the in vivo studies, we assessed protein levels of key readouts from our culture studies. We found that IATL induced a significant increase in caspase-3 activity in tumor tissues (Fig. 7e). In addition, our results showed that IATL treatment significantly increased the level of lipid peroxidation product (MDA), a marker of ROS, in tumor tissues (Fig. 7f). Moreover, IATL treatment increased the expression of CHOP in the mRNA and protein levels (Fig. 7g-i), and decreased the protein expression levels of p-STAT3 and STAT3 in tumor tissues (Fig. 7j-k). Taken together, these results suggest that IATL inhibited tumor growth in vivo by inducing ROS production, which was in accordance with the mechanism in vitro.

## Discussion

The aim of the present study was to elucidate the molecular mechanisms of anti-cancer effects of IATL in prostate cancer cells, and to investigate its effects on the growth of prostate cancer cells in vivo in a subcutaneous xenograft model. We found that IATL inhibited the growth and induced apoptosis in prostate cancer cells. IATL stimulated a rapid increase in reactive oxygen species (ROS) production in prostate cancer cells. By increasing intracellular ROS levels, IATL increased the expression of some ER stress-related proteins in a dose-dependent manner in prostate cancer cells. Importantly, blockage of ROS production significantly reversed IATL-induced ER stress and cell apoptosis. IATL also decreased the protein expression levels of p-STAT3 and STAT3, and the effects of IATL were reversed by pretreatment with NAC. In vivo, administration of IATL (10 mg/kg) reduced both tumor volume and weight, and no significant adverse effects were noticed in the IATL-treated mice. Moreover, treatment of mice with IATL was also associated with induction of ER stress and inhibtion of STAT3 in vivo.

We first noted that IATL reduced the viability and induced apoptosis in two prostate cancer cell lines. These observations are consistent with the previous results obtained by Azhar Rasul et al. who reported that IATL can inhibit proliferation and induce apoptosis in PC-3 and DU145 cells [13]. Reactive oxygen species (ROS) are by-products of aerobic metabolism. Compared with normal cells, many types of cancer cell have increased levels of ROS [22, 23]. Therefore, it might be possible to selectively kill cancer cells by pharmacological ROS insults [24–26]. Some previous studies have shown that ROS production is involved in the biological functions of IATL [9, 12]. In accordance with previous studies, results of our study indicated that IATL induced a rapid increase in ROS production in PC-3 and DU145 cells.

The endoplasmic reticulum (ER) is responsible for folding and processing proteins entering the secretory pathway. Alterations in the ER folding environment cause the accumulation of misfolded proteins in ER lumen can cause ER stress [27]. ER stress is emerging as a modulator of different pathologies and as a critical mechanism contributing to cancer cell death in response to therapeutic agents [28, 29]. It has recently been shown that metformin, tannic acid, and chrysin could induces ER stress-dependent apoptosis in prostate cancer cells [16, 30, 31]. Therefore the therapeutic modulation of the pro-apoptotic ER stress could be a potential strategy for the treatment of prostate cancer. However IATL’s effects on pro-apoptotic ER stress in prostate cancer cells remains unknown. In the present study, we found that IATL treatment induced ER stress response, which is highlighted by elevated levels of p-eIF2α and ATF4, as well as an increase in the level of CHOP. CHOP is considered as an important marker of ER stress-induced apoptosis. Findings presented here demonstrated that the siRNA-mediated knockdown of CHOP markedly inhibited IATL-induced apoptosis in PC-3 cells. Furthermore, our results showed that blockage of ROS production by NAC fully reversed IATL-induced ER stress and cell apoptosis, suggesting ER stress activation and cell apoptosis induced by IATL is dependent on ROS production.

Constitutive STAT3 activation is often displayed by various carcinomas including prostate cancer, and pharmacological drugs that can abrogate deregulated STAT3 activation may have a potential for cancer therapy [32, 33]. Blockage of STAT3 signaling pathway may lead to growth inhibition and apoptosis in cancer cells. We studied the effect of IATL on constitutively active STAT3 in DU145 cells, and found that IATL can suppress STAT3 phosphorylation at Tyr 705 as observed by western blot. IATL also decreased the protein expression of STAT3, and the effects of IATL were reversed by pretreatment with NAC. This study demonstrates a unique approach for targeting STAT3 by ROS-inducing anticancer agents.

## Conclusions

In summary, we investigated the anti-cancer effects and the underlying mechanisms of IATL in prostate cancer cells. We found that IATL reduced the growth of prostate cancer cells through increased production of ROS, activation of ER stress pathway, and inhibition of STAT3. These results indicate that IATL possesses great potential as a promising drug candidate for the treatment of prostate cancer. In addition, our results indicate that ROS production could be targeted for the development of new anti-cancer drugs.

## Abbreviations

ATF4: Activating transcription factor-4
CHOP: C/EBP homologous protein
ER stress: Endoplasmic reticulum stress
IATL: Isoalantolactone
p-eIF2α: Phosphorylation of eukaryotic initiation factor-2α
ROS: Reactive oxygen species
siRNA: Small interfering RNA
STAT3: Signal transducer and activator of transcription 3
